# Yoga of Immortals Intervention Reduces Symptoms of Depression, Insomnia and Anxiety

**DOI:** 10.3389/fpsyt.2021.648029

**Published:** 2021-06-22

**Authors:** Sadhna Verma, James Donovan, Hari S. Tunuguntla, Renuka Tunuguntla, Babu V. Gupta, Ayon Nandi, Ishan Shivanand

**Affiliations:** ^1^University of Cincinnati College of Medicine, Cincinnati, OH, United States; ^2^Rutgers-Robert Wood Johnson Hospital, New Brunswick, NJ, United States; ^3^Internist, Hunterdon Center for Healthy Ageing, Flemington, NJ, United States; ^4^Psychiatrist, Neuropsych Center of Greater Cincinnati, Cincinnati, OH, United States; ^5^Johns Hopkins University School of Medicine, Baltimore, MD, United States; ^6^SYC Infinite, San Francisco, CA, United States

**Keywords:** depression, anxiety, insomia, mindfullness, meditation, mental health, COVID-19

## Abstract

**Background:** Depression, anxiety, and disordered sleep are some common symptoms associated with sub-optimal mental health. During the COVID-19 pandemic, mental health issues have grown increasingly more prevalent in the population. Due to social distancing and other limitations during the pandemic, there is a need for home-based, flexible interventions that can improve mental health. The Yoga of Immortals (YOI) mobile application provides a structured intervention that can be used on any mobile device and applied from the user's home.

**Methods:** A total of 1,505 participants were enrolled in the study and used the YOI app for an 8-week period. Participants were asked to fill out three questionnaires: The Patient Health Questionnaire, 8 items (PHQ-8), the Generalized Anxiety Disorder questionnaire (GAD-7) and the Insomnia Severity Index (ISI). These three items were completed by 1,297 participants a total of four times: before starting YOI, two more times during use, and a fourth time after the 8-week usage period. Changes in PHQ8, GAD7 and ISI in participants were compared to a control group, who did not use the YOI app but completed all questionnaires (590 controls finished all questionnaires).

**Results:** Participants reported significant decreases in depression and anxiety-related symptoms. Compared to baseline, PHQ-8 scores decreased 50% on average after the 8-week period. GAD-7 scores also decreased by 40–50% on average, and ISI scores decreased by 50%. These changes were significantly greater (*p* < 0.05) than that observed in the control group. Participants who reported a previous diagnosis of depression and generalized anxiety reported significantly larger decreases in PHQ-8 and GAD-7 as compared to participants with no prior diagnosis (*p* < 0.05).

**Conclusions:** Regular use of the YOI intervention over an 8-week period led to significant decreases in symptoms of both depression and anxiety, as well as alleviation of insomnia.

## Introduction

Mental health is a public health issue of increasing concern. The World Health Organization (WHO) estimated that one in four people worldwide will be affected by mental disorders at some point over their lifetimes (1). Depression and anxiety are some of the more common psychiatric diagnoses, with estimates for the overall prevalence ranging from 4% up to 20% (2, 3), across various populations.

Both conditions are associated with decreased well-being and social functioning and affect not only the individual but family and friends as well. Further, both depression and anxiety tend to be underrecognized and undertreated (4), adding to the impact of both disorders.

Recently, the COVID-19 pandemic, due to the prolonged impact of physical distancing, stay-at-home orders and the increased mortality and morbidity associated with the disease, has been associated with numerous and increasing mental health challenges. In a poll of over 5,000 US residents conducted by (5), 40% of respondents reported an adverse mental or behavioral health condition. An earlier poll conducted by The Kaiser Family Foundation also found that the pandemic affected the mental health of 56% of adults in the US (6), and similar trends have been reported in other countries (7, 8). Even previous to the pandemic, access to treatments and mental health services was a obstacle for many patients (4). During the pandemic, social distancing and travel restrictions have created additional barriers, not just for professional mental health services, but also in terms of access to social support networks, such as family, friends, social and religious groups. Therefore, there is a need for safe, effective interventions that can be completed from the safety and comfort of an individual's home.

Yoga of Immortals (YOI) is a comprehensive program developed by ShivYog, an organization dedicated to teaching ancient yogic practices. Both mindfulness and meditation-based practices have enjoyed increasing popularity in contemporary psychotherapy (9), as such practices can be part of a “self-help” treatment for anxiety and depression. However, the application of mindfulness meditation in mental health settings has led to the proliferation of studies pervaded by a lack of comprehensive strategy and limited partial applications (10). The complete practices of YOI have, for many centuries, only been passed on through in-person sessions. However, during the pandemic, a structured YOI program was created that can be used on mobile platforms, making these interventions more accessible to a wider audience. The mobile application provides the user with a complete YOI program that can be accessed at any time on one's smartphone or other mobile device. Therefore, the YOI app can deliver interventions and assessment on-demand, overcoming barriers of time, cost and accessibility.

This study is part of an ongoing trial where the goal was to assess and quantify the extent of the benefits that the YOI mobile app program can provide, specifically for symptoms of anxiety, depression, and insomnia.

## Materials and Methods

### Study Population

Study participants were recruited through a link distributed through common social media platforms. Prior to the intervention, all participants were asked to complete the following questionnaires: (1) the Patient Health Questionnaire-8 (PHQ-8), (2) the Generalized Anxiety Disorder 7-item (GAD-7) scale, and (3) the Insomnia Severity Index (ISI). All three are well-validated screening tools, used for population screening of insomnia, symptoms of depression and symptoms of anxiety. Baseline assessment also included demographic variables including age, race/ethnicity, marital and employment status, education, and occupation. Participants were also asked to report if they had a diagnosis of any underlying psychiatric disorder and use of mental health services including psychotherapy and/or use of medications (e.g. antidepressants or other psychotropic medications).

All participants were at least 18 years old and able to read and understand English, as per the inclusion/exclusion criteria. As this study was intended to be an initial pilot study to establish the efficacy of the intervention, and the inclusion/exclusion criteria were purposely broad so as to recruit a broad and diverse study population.

Participants were considered to have completed the study if they responded to all questionnaires over the entire 8-week intervention period. The “final analysis group” consisted of participants and controls that completed all questionnaires.

A total of 1,505 participants enrolled in the study, of which 1,297 participants completed all questionnaires and were included in the final data analysis. The participant group ranged in age from 18–80, with 80% of the participants between 26–58 years old. The study sample has approximately equal numbers of male and female subjects (51% female, 49% male).

A comparison control group was also recruited using the same eligibility criteria. Control subjects also completed the same questionnaires and were provided articles on mental well-being as an intervention strategy. The control group did not use the YOI app during the 8 week study period. In total, 1,300 control subjects were recruited, of which 591 controls completed all questionnaires and were used in the final analysis group. The control group had equivalent demographic characteristics as the participant group.

We also tested if any demographic variables changed significantly between the initial recruited group and the final set of participants and controls that completed the entire study. For both participants and controls, the mean age did not significantly differ between the initial recruited pool and the final set of those who completed the study; age range and percentage of male vs. female also did not significantly differ between the two sets.

### Intervention

Participants were asked to complete the intervention using the YOI app, which consisted of 112 sessions over the course of 8 weeks, with different sessions each week. Participants were asked to read and watch the associated instruction in preparation for the weekly sessions, which lasted for 30 min each. The intervention included two sessions per day – one in the morning and one in the evening. Participants completed the intervention during the study period of April to June 2020.

The two sessions focused on different techniques: morning sessions included a combination of whole-body movements, postures, and yogic breathwork (cyclical controlled breathing practices including abdominal breathing) synchronized with meditation and chants.

The evening sessions consisted of slow yet deep yogic breathwork and meditations. Each week, the sessions became more advanced, by building upon the work of prior weeks.

After completion of the last session, participants were asked to complete all screening questionnaires for a fourth and final time, and also to complete their study surveys. To ensure data quality, human verification and attention checks were implemented throughout the survey; the data was further inspected visually for response irregularities suspicious for bots.

Participants were given access to the YOI app via a download link, and only participants (not controls) were given the link. Thus, control subjects had no access to the YOI intervention. However, controls were questioned if they performed any other yoga or mindfulness practices during the intervention period. Controls that did so were excluded from the final analyzed control data.

### Assessment Scales

Symptoms of depression were assessed using the 8-item version of the Patient Health Questionnaire (PHQ-8, (11)). The PHQ-8 versions used was the standardized and modified response set of (12) (A four-point Likert scale from 0 to 3; 0 to 1 day = “not at all,” 2 to 6 days = “several days,” 7 to 11 days = “more than half the days,” and 12 to 14 days = “nearly every day,”). The scores for each item were summed to produce a total score between 0 and 24 points. The ranges of total scores are categorized as follows: 0 to 4 = no significant depressive symptoms; 5 to 9 = mild depressive symptoms; 10 to 14 = moderate; 15 to 19 = moderately severe; and 20 to 24 = severe. Current depression symptoms were defined as a PHQ-8 score of ≥10 (13) which, regardless of diagnostic status, typically correlate with clinically significant depression (12, 14).

Symptoms of anxiety were assessed using the 7-item Generalized Anxiety Disorder scale (GAD-7, 21). The GAD-7 consists of 7 items which asks patients to indicate how often they have been “bothered” by the specific symptoms listed in the questionnaire. Responses are scored on a four-point Likert scale ranging from 0 (not al all) to 3 (“Nearly every day”). Individual scores were then summed for the total score, which ranged from 0 to 21 points. Similar to the PHQ-8, cut points of 5, 10, and 15 were interpreted as representing mild, moderate, and severe levels of anxiety; these cut points previously demonstrated high sensitivity and specificity for symptoms of anxiety (15).

Insomnia severity was assessed using the Insomnia Severity Index (ISI), an instrument posing seven questions to assess current (i.e., preceding 2 weeks) sleep characteristics. The first three items pose questions related to sleep onset, sleep maintenance, and early morning awakening. Subsequent items assess the degree of satisfaction or dissatisfaction with the current sleep pattern, how the current sleep pattern interferes with daily functioning, how noticeable the impairment attributed to the sleep problem is, and how worrisome the current sleep problem is. Items were rated on a five-point Likert scale (“0” representing none or not at all and “4” representing very much). Total scores ranged from 0 to 28, with higher combined scores indicating worse insomnia severity (16). Participants were placed in total score groups as follows: 0–7 = no clinically significant insomnia; 8–14 = subthreshold insomnia; 15–21 = clinically significant insomnia (moderate); 22–28 = clinically significant insomnia (severe).

### Statistical Analysis

All statistical analysis was done using the Statistical Package for Social Science (SPSS). For demographic parameters, descriptive statistics were used to express results as numbers and percentages. As all three scales were based on Likert-like scales and then summed, the total scores were tested for the assumption of normality (*p* > 0.1, Shapiro-Wilk test), and treated as normally distributed interval data (17). The ISI, PHQ8 and GAD-7 data were expressed as mean ± SD (standard deviation). Demographic and other variables used in the analysis were also tested for normality (*p* > 0.1, Shapiro-Wilk test).

In a previous initial and exploratory analysis used, paired *t* tests were done for within group comparison. A two-sided *p*-value < 0.05 was considered statistically significant. Chi-squared test was applied for comparison of percentage scores between pre and post YOI intervention scales. A *p* value < 0.05 was considered statistically significant. This exploratory analysis was used to test for overall trends in the data.

To robustly test for changes in mean scores as well as subitem scores across grouping factors, we first used two-way ANOVA to identify factors which significantly contributed to between group differences (participant vs. control) and within group differences (pre vs. post intervention). The normalized mean scores or means for each subitem score served as the main dependent variable. *Post-hoc* tests using Tukey's honest standardized differences method to correct for multiple comparisons were used to compare across each level of both factors following the initial ANOVA. As with the previous analyses, the threshold was set at *p* < 0.05.

The ANOVA analysis identified several potential significant predictors of final scores. We wished to test how these factors interacted with each other and affected the variability of all three sets of scores. Therefore, we used regression analysis, with the normalized mean score as the dependent variable, with age and gender as covariates.

### An Institutional Review Board Approval

The study was approved by the Institutional Review Board, University of Cincinnati. Informed consent was obtained from all participants included in this study.

## Results

In terms of demographic makeup, both control and participant groups included subjects of both genders and a wide range of ages [26–85] but concentrated between 30–58 years (Table 1). The distribution of age, gender and race did not differ between the controls and participants (Pearson's c^2^ > 3, *p* > 0.1). In addition, for both controls and participants, there were also no significant differences between the recruited sample and the analyzed sample of completers (Pearson's c^2^ > 3, *p* > 0.1).

**Table 1:** Demographic characteristics of the study participants and controls.

**Demographics**		**Participants**		**Controls**	
		***N*** **(%)**		***N* (%)**	***N* (%)**
		**Recruited**	**Completed**	**Recruited**	**Completed**
Age	<18–25	93 (6.2%)	80 (6.2%)	130 (10%)	65 (11%)
	26–36	455 (30.3%)	394 (30.4%)	403 (31%)	177 (30%)
	37–47	461 (30.7%)	396 (30.5%)	455 (35%)	213 (36%)
	48–58	304 (23.4%)	304 (23.4%)	235 (18.1%)	118 (20%)
	59–69	113 (8.7%)	113 (8.7%)	65 (3%)	17 (3%)
	70–80	9 (0.7%)	9 (0.7%)	10 (0.8%)	0
	>80	1 (0.08%)	1 (0.08%)	1 (0.1%)	1 (0.08%)
Gender	Male	770 (51.2%)	765 (50.1%)	676 (52%)	301 (51%)
	Female	735 (48.8%)	729 (48.5%)	624 (48%)	290 (49%)
Race/Ethnicity	Asian	1,150 (76.4%)	1,142 (88.1%)	1,114 (88%)	526 (89%)
	White	9 (0.7%)	9 (0.7%)	10 (0.8%)	9 (0.7%)
	Other	135 (10.4%)	135 (10.4%)	135 (10.4%)	56 (9.4%)

### Depression Symptoms (Patient Health Questionnaire-8)

**Figure 1:**
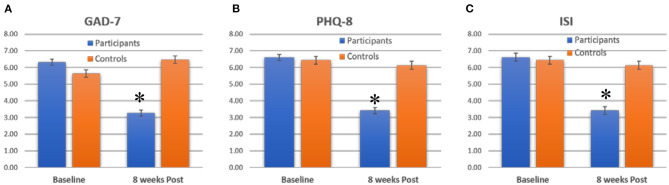
Average scores for **(A)** GAD-7, **(B)** PHQ-8 and **(C)** ISI for participants and controls, at baseline and 8 weeks post. Bars show mean +/–standard error. *indicate significant decreases.

At baseline, both the control group and the participants had similar PHQ-8 scores: Controls 6.4+/5.6, Participants 6.61+/−5.9 (Mean +/-SD). However, after the intervention period, participants scores dropped to 3.41+/−4.44, while in controls the scores remained similar (6.1+/−5.5). On average, PHQ-8 scores dropped an average of 3.31+/−4.93 across all participants (Figure 1A). Subitem scores also dropped significantly, by >50% in all cases. The initial exploratory analysis found that both the decreases in PHQ-9 totals and subitems were significant in participants (paired *t*-test, *p* < 0.05) but not in controls (paired *t*-test, *p* > 0.5). When the PHQ-8 scores in participants was compared to the change in controls by two-way ANOVA, both group status (df = 1, *F* = 5.1, *p* < 0.05) and time (df = 1, *F* = 12.3, *p* < 0.05) were factors that significantly affected the score. *Post-hoc* tests showed that participants had a significant decrease in scores as compared to controls – which was tested by comparing the pre vs. post level of the time factor in participants (*post-hoc* test, *p* < 0.01, effect size *d* = 0.8).

### Anxiety Symptoms (Generalized Anxiety Disorder Scale-7)

At baseline, both the control group and the participants had similar GAD-7 scores: Controls 5.6+/5.2, Participants 5.61+/−5.9 (Mean+/-SD). However, after the intervention period, participants scores dropped to 3.41+/−4.44, while in controls the scores remained similar (5.8+/−6.4). On average, GAD-7 scores dropped an average of 2.2+/−4.5 across all participants (Figure 1B). Subitem scores also dropped significantly, by >50% in all cases. Both the decreases in GAD-7 totals and subitems were significant in participants (paired *t*-test, *p* < 0.05) but not in controls (paired *t*-test, *p* > 0.5). When the GAD-7 scores in participants was compared to the change in controls by two-way ANOVA, both group status (df = 1, *F* = 4.2, *p* < 0.05) and time (df = 1, *F* = 10.1, *p* < 0.01) were factors that significantly affected the score. *Post-hoc* tests showed that participants had a significant decrease in scores as compared to controls – which was tested by comparing the pre vs. post level of the time factor in participants (*post-hoc* test, *p* < 0.01, effect size *d* = 0.64).

### Insomnia Symptoms (Insomnia Severity Index)

Similar to the GAD-7 and PHQ-8 results, controls and participants had similar ISI scores at baseline (Controls 6.5+/−7, Participants 7.6+/−7.6). After the intervention period, participants reported a 50% decrease in ISI scores, dropping to 3.31+/−4.94 (Figure 1C). In contrast, controls did not show any significant change in ISI scores post intervention (6.4+/−6.6). The decrease in participants was significant (paired *t*-test, *p* < 0.05), but the decrease in controls was not (*p* > 0.1, paired *t*-test). Using two-way ANOVA, both group status (df = 1, *F* = 3.1, *p* < 0.05) and time (df = 1, *F* = 9.2, *p* < 0.01) were factors that significantly affected the score. *Post-hoc* tests showed that participants had a significant decrease in scores as compared to controls – which was tested by comparing the pre vs. post level of the time factor in participants (*post-hoc* test, *p* < 0.01, effect size *d* = 0.54).

### Regression Analysis

**Figure 2:**
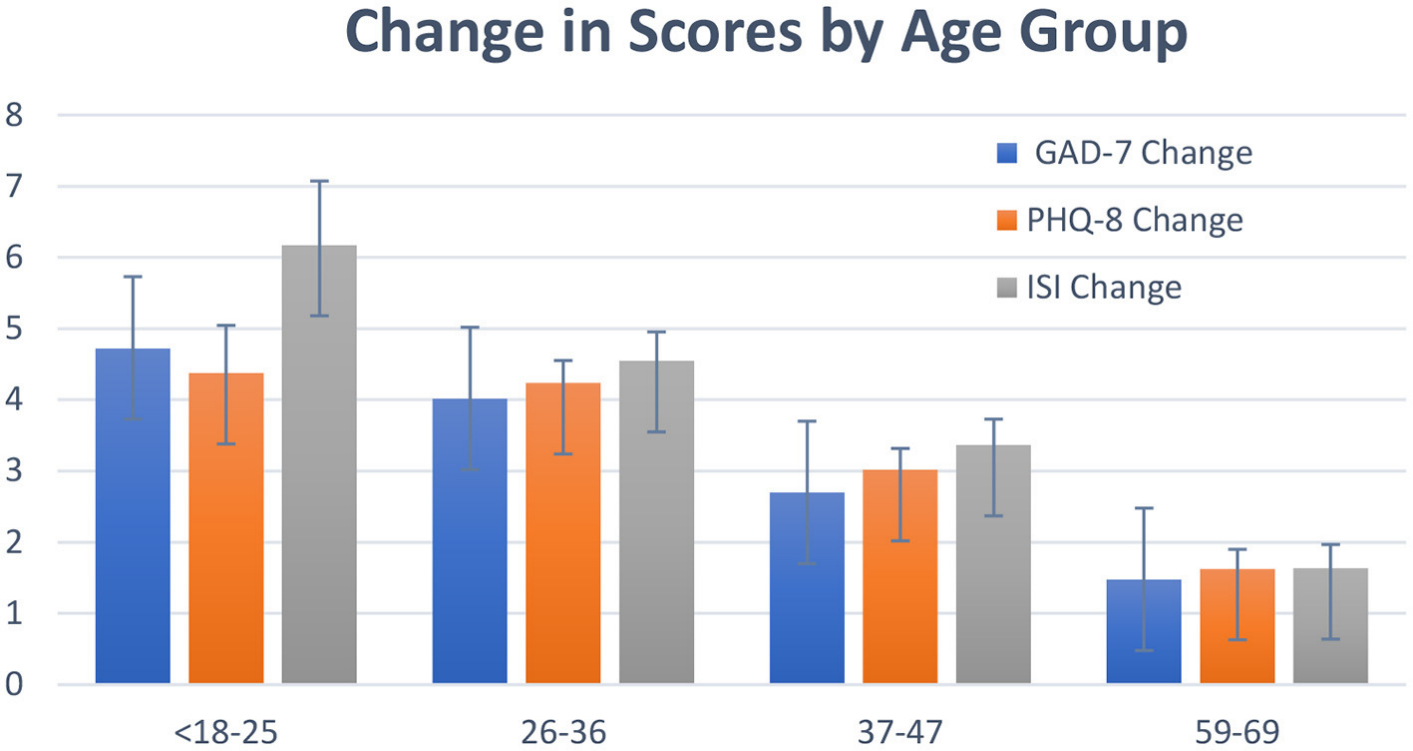
Change in GAD-7, PHQ-8 and ISI for participants, by age group. Bars show mean +/–SE of the total change in scores for each group.

**Figure 3:**
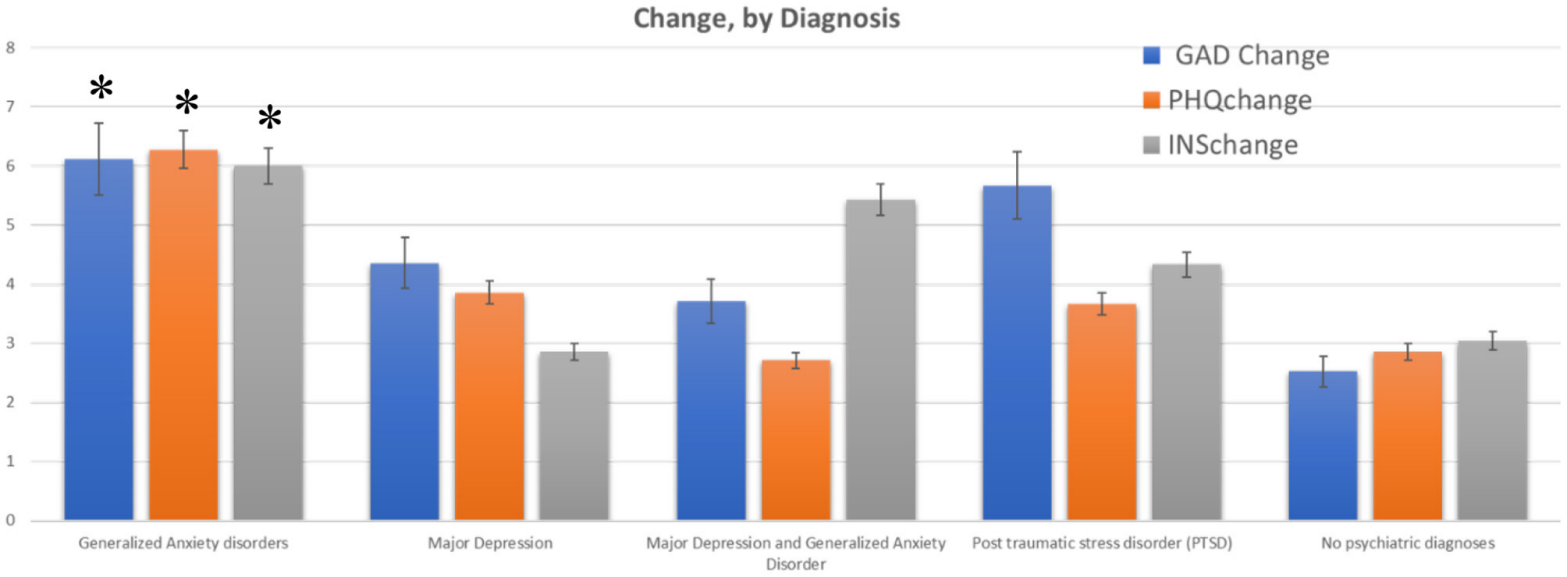
Change in GAD-7, PHQ-8 and ISI for participants with self-reported mental health diagnoses. Bars show mean +/–SE of the total change in scores for participants who indicated (by self-report) that they had a current psychiatric diagnosis (listed on the x-axis. One group reported having both major depression and generalized anxiety disorder). A comparison group of subjects with no reported diagnoses showed a much lower change across all scores. *indicate the changes that were significantly different from the control (no psychiatric disorders) group.

From the regression analysis, several other key results were found for specific groups across all three scales. First, younger participants (aged 26–36 and 37–47) had significantly greater decreases in PHQ8, GAD7 and Insomnia, when compared to all other age groups (*post hoc* tests comparing levels of factor age group and pre-post, *p* < 0.05, df = 2, effect size = 0.72) (Figure 2). Subjects who had self-reported diagnoses of Generalized Anxiety had significantly higher decreases in GAD-7 (*p* < 0.01, comparing to subjects with no Anxiety diagnosis) (Figure 3). Subjects who reported having diagnoses of both Depression and Anxiety had significantly higher improvements in PHQ8 and Insomnia (*post hoc* tests comparing levels of diagnostic group and pre-post, *p* < 0.05, df = 2, effect size = 0.52) comparing to subjects without either diagnosis (Figure 3).

**Figure 4:**
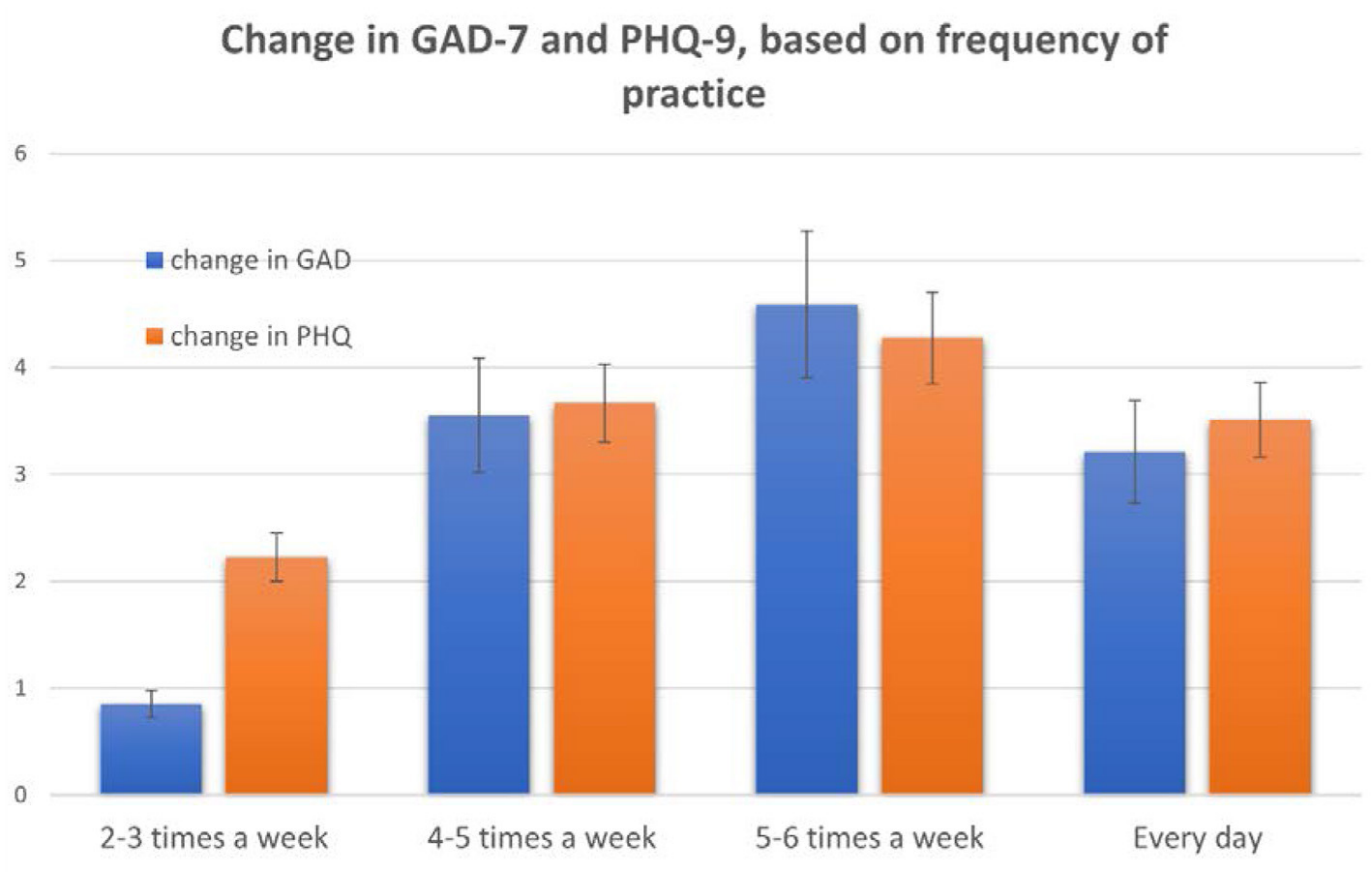
Changes in GAD-7 and PHQ-8 based on how often participants reported using the YOI module via the mobile app. These results are based on answers to the question “How often do you practice the YOI module in the morning?” Bars show mean +/–SE.

Regression analysis also identified frequency of practice, (“How often did you practice YOI?”) as a highly significant factor affecting GAD-7 [estimated *b* = 1.2, 95% CI (0.5, 3.5), *p* < 0.001] and PHQ-8 [estimated *b* = 1.4, 95% CI (0.3, 3.8), *p* < 0.001]. Frequency of practice did not significantly affect insomnia scores, however. *Post-hoc* tests following an ANOVA with the factor of pre vs. post and frequency of practice indicated that subjects who reported practicing the module 4–6 times per week had better results [significantly higher decreases across both scales when comparing to subjects who reported practicing 1–2 times per week: estimated mean difference PHQ-8 = 3.5+/−2.1, *p* < 0.05; estimated mean difference GAD-7 = 3.2+/−3.5, *p* < 0.05 (Figure 4)].

## Discussion

Overall, participants who completed the YOI intervention reported significant decreases in symptoms of anxiety, depression, and insomnia. This decrease is comparable to that reported for cognitive behavioral therapy for depression and anxiety (18–22) – such approaches report effect sizes in the range of Cohen's *d* = 0.5 to 0.8. In terms of depression, the YOI intervention had an effect size of *d* = 0.4 to 0.6. However, results from studies of cognitive-behavioral therapy are largely based on longitudinal studies which follow patients for several months. To directly compare the YOI intervention to these other treatments, participants should be followed for longer periods of time.

### Depression Symptoms

**Figure 5:**
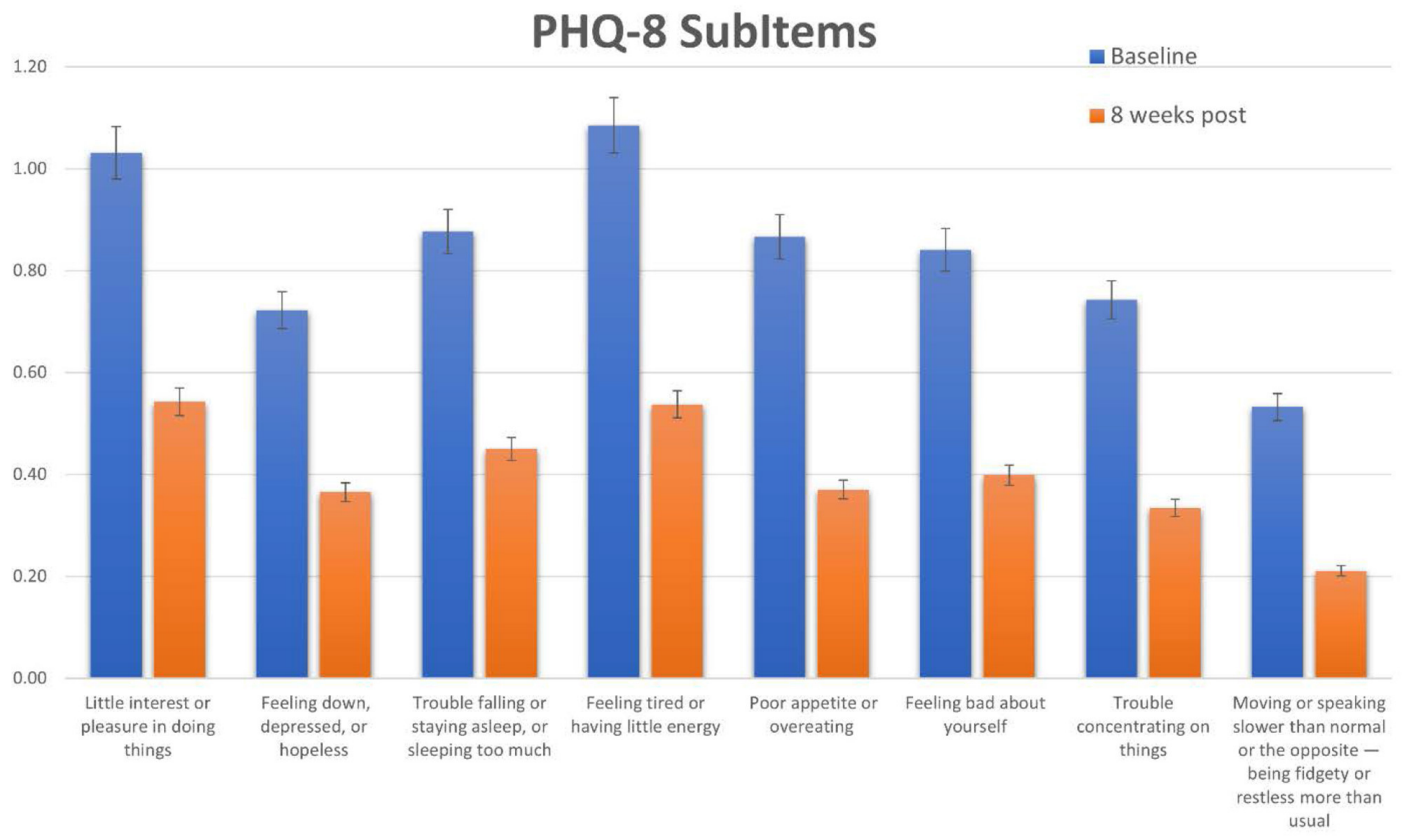
Scores for each subitem of the PHQ-8. Specific scores and baseline and 8 weeks post for each PHQ-8 subitem question.

Participants reported significant decreases for total PHQ-8 scores, as well as significant decreases in specific symptoms. Specifically, the largest decrease was seen for the item “Feeling down, depressed, or hopeless,” followed by the items for “Trouble falling asleep” and “Feeling tired or having little energy.” (Figure 5) Overall, the decrease in symptoms seen with the YOI intervention are comparable to those seen in studies of exercise-based interventions (23) and slightly lower than that reported in a recent meta-analysis of cognitive-behavioral therapy (24). Exercise based interventions reported an effect size of Cohen's *d* = 0.5 to 0.6 when using the PHQ-8 to measure symptoms, while CBT yielded effect sizes of *d* = 0.6 to 0.81, also using the PHQ-8. The results of this study had an effect size of 0.5 to 0.6, comparable to both exercise-based and CBT interventions.

Specific subgroups of patients had slightly better results with the app in terms of reducing depression symptoms, specifically, younger participants and those who reported a diagnosis of depression. This result indicates that the YOI intervention may be of specific benefit to those already suffering from depression. The sub-item results indicate that the intervention had a slightly larger effect on overall mood and energy levels than other aspects of depressive symptoms.

### Anxiety Symptoms

**Figure 6:**
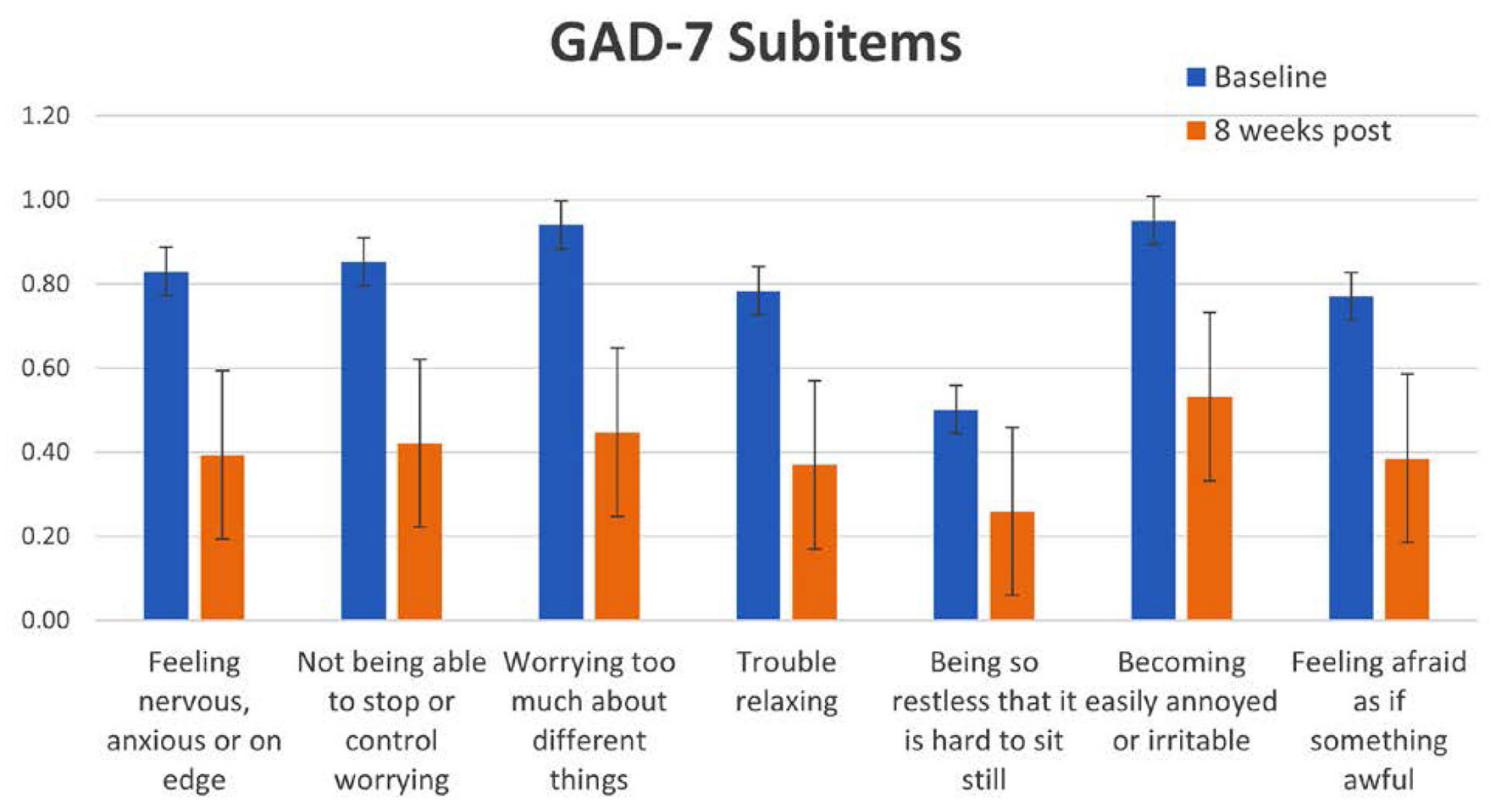
Specific scores and baseline and 8 weeks post for each GAD-7 subitem question.

In line with the results with depressive symptoms, GAD-7 total scores decreased significantly as well as specific subitems. The largest decreases were seen in the items related to “Feeling nervous, anxious or on edge,” “Worrying too much” and “Trouble relaxing” (Figure 6). For the reductions in GAD-7 the effect size in the current study was Cohen's *d* = 0.6 to 0.7. The results with GAD-7 are, again, comparable to those seen from exercise-based interventions [Cohen's d for exercise = 0.5–0.6, (25)]. However, for anxiety, the YOI intervention performed slightly better than CBT for anxiety [*d* = 0.3 – 0.56, (26)].

Participants that had a pre-existing diagnosis of generalized anxiety disorder reported better results with the YOI intervention than those who did not. Once again, this was similar to the effects seen for PHQ-8 and depression. There is a high degree of comorbidity between depression and anxiety (27), and the two disorders share several genetic and non-genetic risk factors. Furthermore, the presence of comorbid anxiety disorder is highly associated with worse outcomes for depression and other mental health disorders (28). Therefore, the fact that the YOI intervention targets both depressive and anxiety-related symptoms simultaneously, it can potentially provide specific improvement for those patients with these comorbidities.

### Insomnia Symptoms

**Figure 7:**
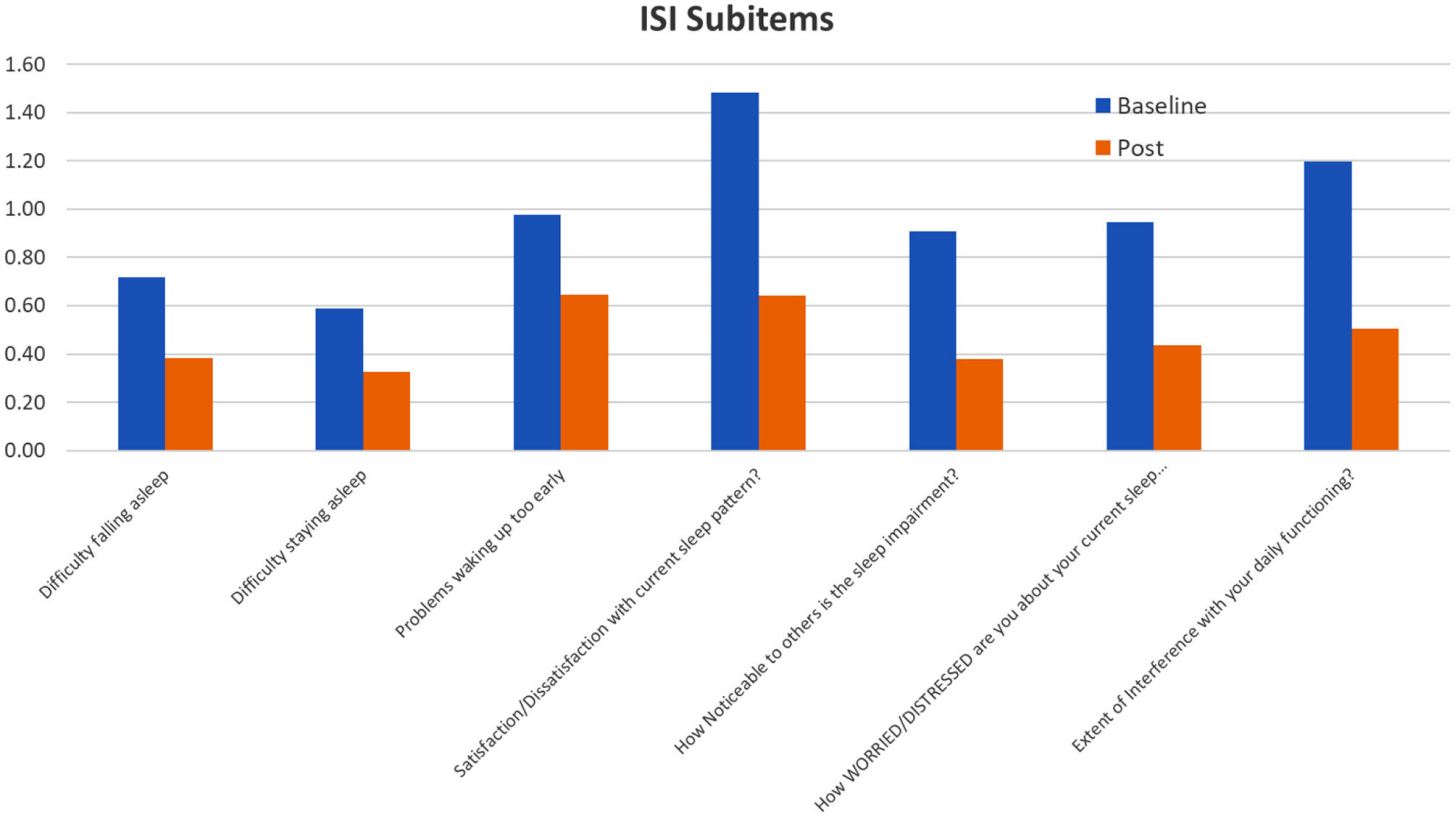
Specific scores and baseline and 8 weeks post for each ISI subitem question.

Insomnia symptoms, which were measured by the ISI questionnaire, also showed ~50% decrease in terms of the total score as well as across all subitems (Figure 7). Unlike with depression and anxiety, all ISI subitems had equivalent changes. Since both depression and anxiety can have significant effects on sleep quality (29) it is not surprising that insomnia symptoms also decreased along with GAD7 and PHQ8 scores.

The relationship between insomnia and depression/anxiety may be bi-directional, however, as recent studies have found that insomnia can impair emotional regulation (30). Disordered sleep has been shown to lead to heightened emotional reactivity, which can exacerbate symptoms of depression and anxiety. Conversely, good sleep quality is associated with higher positive emotions, which can help reduce symptoms (30). Additionally, sleep disturbances during insomnia can increase the risk of inflammatory disorders and a weakened immune system (31). Ensuring high quality sleep, therefore, can improve not only mental health symptoms but can also immunity.

### Overall Changes

The connections between depression, anxiety, insomnia, emotional regulation and immunity have been the subject of a growing field of research (32). Several studies have found that depressive symptoms can mediate the effects of immunologically based diseases. The relationship between anxiety and depression (28) and the link between insomnia and immunity (31) could potentially also play a role in the links between mental health, sleep quality, and immunity (33). Therefore, in the context of the current global pandemic, there is an urgent need to provide effective, accessible solutions that can improve mental health and sleep quality. Improving those symptoms may in turn have a positive effect on immune system health.

Using the YOI app resulted in decreases across all three scales used in this study, suggesting that the intervention can improve symptoms across multiple areas. For depression, PHQ-8 scores dropped an average of 3.3 points for participants, which is a clinically significant decrease, as a drop of 3 points can represent a shift for “moderate” to “mild” symptoms, or “mild” to none. Similarly, the decrease in GAD-7 was also clinically significant. Using the YOI app thus improved symptoms in all 3 areas improved symptoms statistically and clinically significant amount.

## Limitations and Future Directions

The results of this study were based on self-reported questionnaires, which have an inherent bias, as the subjects may exaggerate or minimize specific symptoms. However, such self-reported questionnaires are routinely used in psychology studies and are well-validated instruments. The study did rely on participant's willingness to adhere to the intervention and fill out all questions. Thus, there may have been a bias to the results as participants who had better results may have been more inclined to continue to participate in the study. The use of a control group reduced this bias, however.

Certain information could not be obtained from the self-report surveys during this initial study. The major limitation was that the study was conducted during the initial shutdowns of the COVID-19 pandemic in April-June of 2020. Therefore, self-reported questionnaires only could be used. Relying on self-report is a potential bias in this study as participants may not have provided complete and accurate information about their health status. For the information on disease diagnosis, this information was obtained solely from the survey responses, no other confirmation of diagnoses was done. Participants were instructed to list any specific psychiatric diagnoses they had received from their doctors. Nevertheless, the aim of this study was to show efficacy in a broad population, which is demonstrated by the consistent reduction in self-reported symptoms. Further, the regression analysis was able to explicitly test the effect of these specific variables (presence of psychiatric diagnoses and other health conditions).

Additionally, the surveys did not ask for weight or BMI information, or details on sleep hygiene. High BMI can lead to sleep apnea and poor sleep hygiene contribute to disrupted sleep, and these factors, if present in our study population, could have affected the results. Further studies using the YOI intervention are ongoing and so future studies will strive to collect more detailed and nuanced information.

A possible future direction will be to compare YOI with other yoga mindfulness practices. However, YOI is a unique combination of yoga posts, breathing exercise and meditation. Thus, a challenge will be to find appropriate interventions to compare to. Nevertheless, YOI could be compared to other interventions that involve physical activity, to see if the specific type of physical activity indicated by YOI contributes to the effect seen on depression and anxiety scores.

The current results reported here are part of an ongoing study, which will track participants for up to 6 months of using the YOI app. The future data will hopefully confirm that the results seen so far are continued, and if continued use of the YOI app can act as a preventative measure for depression, anxiety and insomnia symptoms.

## Conclusions

Overall, the results of the current study show a significant, consistent reduction of symptoms related to depression, anxiety and insomnia. Participants who already reported suffering from anxiety and/or depression reported slightly greater benefits from the intervention.

## Data Availability Statement

The raw data supporting the conclusions of this article will be made available by the authors, without undue reservation.

## Ethics Statement

The studies involving human participants were reviewed and approved by University of Cincinnati. The patients/participants provided their written informed consent to participate in this study.

## Author's Note

This study is performed by a team of physicians and researchers. This team has a keen interest in scientific research to validate the efficacies of complementary and alternative treatment modalities. This research study is an attempt to verify the effectiveness of Yoga of immortals as a novel intervention strategy for insomnia and depression.

## Author Contributions

SV, JD, HT, RT, BG, and IS were responsible for the planning, implementation and data collection and analysis of the study. SV and AN performed data analysis and writing of the manuscript. All authors contributed to the article and approved the submitted version.

## Conflict of Interest

IS was employed by the company SYC Infinite. SYC Infinite did not provide any funding for this study and had no role in the study. The remaining authors declare that the research was conducted in the absence of any commercial or financial relationships that could be construed as a potential conflict of interest.
